# Investigating the Impact of AI-Based Conversational Models as Learning Companions Among Second-Year MBBS Students

**DOI:** 10.7759/cureus.97899

**Published:** 2025-11-26

**Authors:** Karthika Padmavathy, Swathi Sridharan, Chittathur Vignesh, Sivagama Sundari C S, Divya G

**Affiliations:** 1 Pathology, Sri Lalithambigai Medical College and Hospital, Dr. M.G.R. Educational and Research Institute, Chennai, IND

**Keywords:** ai in medical education, chatbot learning, comparative study, pathology education, technology-enhanced learning

## Abstract

The methods of medical education are gradually shifting, with increasing interest in alternative methods that support more interactive and student-centered learning. The aim of this study is to evaluate the effectiveness of a structured digital dialog tool designed to aid second-year undergraduate (UG) medical students in understanding selected topics in pathology. A total of 119 participants were enrolled and divided into two groups: one group engaged with the digital learning module, while the other followed a conventional textbook-based study. The modules were carefully created in consultation with subject experts to ensure alignment with curricular standards. After the study period was over, essays, short-answer questions, and multiple-choice tests were used to assess how well the UG students understood the material. The results showed that students who used textbooks performed better on all types of tests. Still, feedback from the digital group showed that they were very satisfied with the tool, with students saying it made the content easier to understand and more interesting. Although the traditional approach led to better academic performance, the digital method offered clear advantages in terms of learner interest and accessibility. The findings support the idea that combining structured digital support with established teaching methods may enhance both motivation and academic success in medical training.

## Introduction

Plain language summary

This study looked at how well second-year medical students understood important pathology topics when they were taught in two different ways: one group used a digital conversation tool, and the other used regular textbooks. The textbook group performed better on written tests, but many students who used the digital method said it helped them understand the material better and made studying more enjoyable. These results suggest that students may learn better and stay motivated if they use both digital and traditional methods at the same time.

Introduction

As the world of education is changing quickly, we need to find new ways to teach that work with how students learn today [1]. Medical education, in particular, needs approaches that not only give students a solid base of knowledge but also get them involved. As students spend more time on the internet, they want resources that are more interactive, easy to find, and accessible at their own pace.

Among the many developments in educational technology, the use of digital dialogue-based learning tools has gained attention [2]. These tools act like teachers, as they help students ask questions and obtain answers in a style that is organized and conversational [3]. These platforms may help students strengthen their basic ideas, answer queries in real time, and encourage them to study on their own [4].

Even though these new concepts are interesting, the conventional method of using textbooks is still an important tool for medical training [4]. Textbooks remain a reliable way to study because they are structured, comprehensive, and aligned with test requirements [5,6]. But students often look for extra resources that can help them understand difficult subjects or provide a new point of view [7].

This study was undertaken to assess how second-year MBBS students responded to a newly developed digital conversation tool for learning pathology. It aimed to compare not only their academic performance but also their overall learning experience with those who relied solely on textbook-based preparation.

## Materials and methods

This study was a cross-sectional study that examined the academic setting of Sri Lalithambigai Medical College and Hospital (SLMCH) in Chennai. The purpose was to compare and analyze the academic results and learning experiences of second-year medical undergraduate students who learned pathology in two different ways.

Participants were chosen based on their willingness to take part. We included students who gave written consent after receiving full information about the study and were available during the study period. Students who declined to participate or were absent during the intervention and evaluation phases were excluded.

There were 119 students in the study, all of whom were in their second professional year in SLMCH (Phase II MBBS). We divided these students into two groups randomly using a simple lottery method by independent administrative personnel, and both groups were given an equal supervised study duration of 90 minutes to ensure consistent exposure. There were 60 students in Group A who received a structured digital educational module designed to simulate a guided conversation with pathology content built in. Group B, comprising 59 students, studied the same subject matter using the conventional method of textbook reading. The reference textbook for Group B was the most recent edition of Robbins and Cotran Pathologic Basis of Disease, the standard recommended text for pathology in the MBBS curriculum [8].

Before data collection began, the study protocol was submitted to the Institutional Ethics Committee (IEC) of SLMCH and received full ethical clearance. The approval process ensured that all elements of the study complied with institutional and national guidelines for research involving human participants [9,10]. Students were given an in-depth explanation of the objectives, characteristics, and requirements of the research project. They were assured that their academic status would not be affected by their decision to participate or withdraw at any point, and participation was entirely optional. We ensured the confidentiality of student information with great care.

The instructional content for both groups included selected key topics from general pathology. Both groups were given the same duration to study the material. Following the learning period, students were evaluated using a structured assessment that included essay-type questions, short-answer questions, and multiple-choice questions (MCQs). This allowed for a broad assessment of knowledge, analytical thinking, and recall. The evaluation pattern was standardized across both groups to ensure fairness and comparability of results.

Intervention

As part of the educational assistance, two interactive digital learning modules were specifically built. To help students learn essential concepts in pathology, these modules were developed to provide structured support. The topics selected for these modules focused on subjects that are frequently viewed as challenging by first-year medical students. Three senior faculty members from the Department of Pathology contributed to the development of the material.

The modules were designed to simulate a guided conversation between a teacher and a student, arranged in a conversational format [11-13]. This method was chosen to foster active participation, simplify complicated concepts, and provide a learning environment that is more conducive to understanding. The educational content consisted of explanations, examples, and questions designed to stimulate critical evaluation. The modules were made available to students through a user-friendly digital platform accessible from personal devices during the allocated study period [14].

Students in Group A were instructed to use these modules exclusively to prepare for the assigned topics. Meanwhile, students in Group B followed a traditional self-study approach, relying on the most recent edition of Robbins and Cotran Pathologic Basis of Disease. Both groups were given equal time to review the material before being assessed.

## Results

Table 1 and Table 2 show the evaluation results of students on essays, short answers, and MCQs. For the first group (Table 1), the median essay score (out of 20) was 11.00 (IQR: 9.00-13.00), the short-answer score (out of 15) was 9.00 (IQR: 7.00-10.00), and the MCQ score (out of 15) was 12.00 (IQR: 11.00-14.00). The total score (out of 50) was 32.00 (IQR: 29.00-34.00), resulting in an overall percentage of 64.00 (IQR: 58.00-68.00).

**Table 1:** Distribution of assessment scores in the AI (AI-based module) group Distribution of assessment scores in the AI learning companion group, showing the median, 25th percentile, and 75th percentile for essay, short-answer, and MCQ components, along with the total score (out of 50) and overall percentage. MCQ, multiple-choice questions

	Median	Percentile 25	Percentile 75
AI module essay (out of 20)	11.00	9.00	13.00
AI module short answers (out of 15)	9.00	7.00	10.00
AI module MCQ (out of 15)	12.00	11.00	14.00
Total (out of 50)	32.00	29.00	34.00
Overall AI module marks (%)	64.00	58.00	68.00

**Table 2:** Distribution of assessment scores in the TB method group Distribution of assessment scores in the traditional textbook-based (TB) group, showing the median, 25th percentile, and 75th percentile for essay, short answer, and MCQ components, along with the total score (out of 50) and overall percentage. TB, traditional textbook-based; MCQ, multiple-choice questions

	Median	Percentile 25	Percentile 75
TB method essay (out of 20)	15.00	13.00	17.00
TB method short answer	12.00	11.00	13.00
TB method MCQ	9.00	7.00	11.00
Total	36.00	33.00	39.00
TB method marks (%)	72.00	66.00	78.00

The second group (Table 2) scored better. Their median essay score (out of 20) was 15.00 (IQR: 13.00-17.00), the short-answer score was 12.00 (out of 15, IQR: 11.00-13.00), and the MCQ score (out of 15) was 9.00 (IQR: 7.00-11.00). The total score (out of 50) was 36.00 (IQR: 33.00-39.00), with an overall percentage of 72.00 (IQR: 66.00-78.00).

The academic performance of both Group A and Group B students was evaluated using three types of assessments: essay questions, short-answer questions, and MCQs. The scores were analyzed and compared using the Mann-Whitney U test, as the data did not follow a normal distribution [15,16]. A p-value of less than 0.001 across all categories confirmed that the differences observed were statistically significant.

In terms of overall performance, the students in the textbook group scored noticeably higher. Their median score was 72%, with scores ranging from the 25th percentile (66%) to the 75th percentile (78%). In contrast, the digital group recorded a lower median of 64%, with a range of 58% to 68%.

When analyzed by question type, the differences were even more distinct. In essay questions, students using textbooks achieved a median of 75%, compared to 55% in the digital group. A similar pattern was seen in the short-answer questions, with the textbook group achieving a median score of 80%, while the other group achieved only 60%. Notably, the digital group outperformed their counterparts only in MCQs. The group using the digital learning module achieved a median score of 80% in the multiple-choice section. In contrast, the textbook group had a lower median score of 60% in the same category (Table 3).

**Table 3:** Comparison of median scores and IQR between the AI-based module and traditional textbook group across different assessment formats, highlighting statistically significant differences (P<0.0001) Values are expressed as median (25th-75th percentile); P-value determined by Mann-Whitney U test. P<0.05 was considered statistically significant.

	AI as a conversational module			Traditional textbook group			P-value
	Median	Percentile 25	Percentile 75	Median	Percentile 25	Percentile 75	
Overall marks (%)	64.00	58.00	68.00	72.00	66.00	78.00	<0.0001
Essay (%)	55.00	45.00	65.00	75.00	65.00	85.00	<0.0001
Short answer (%)	60.00	47.00	67.00	80.00	73.00	87.00	<0.0001
MCQ (%)	80.00	73.00	93.00	60.00	47.00	73.00	<0.0001

Students who used the digital module shared positive feedback about their experience (Table 4). Many expressed appreciation for the simplicity of the structure, the clarity of the material provided, and the ability to revisit and study the topics at their own speed [17]. Several students noted that the conversational approach created a more engaging learning experience. However, some observed that the content was not as extensive or complete as what they found in standard textbooks.

**Table 4:** Student feedback on the structured digital module (n=119) Data are presented as percentages (number of students).

Feedback statement	Strongly agree (%)	Agree (%)	Neutral (%)	Disagree (%)	Strongly disagree (%)
The structured flow made the content easy to follow	70.6 (84)	24.4 (29)	3.4 (4)	1.7 (2)	0 (0)
The module helped me think through the topic step by step	58.0 (69)	33.6 (40)	6.7 (8)	1.7 (2)	0 (0)
The questions in the module stimulated critical thinking	49.6 (59)	36.1 (43)	10.1 (12)	4.2 (5)	0 (0)
I was more engaged with this format than regular self-study	61.3 (73)	28.6 (34)	5.0 (6)	5.0 (6)	0 (0)

Taken together, these observations suggest an important pattern. While traditional textbooks continue to play a crucial role in helping students perform well in written exams, structured digital learning tools can still offer meaningful support [18,19] (Table 5). They appear to be particularly helpful in boosting interest and confidence, especially when preparing for objective-style assessments like MCQs [20,21].

**Table 5:** Student preferences for learning methodologies (n=119) Data are presented as the number of students and corresponding percentages.

Preferred teaching method	Number of students	Percentage (%)
Structured digital conversational module	81	68.0
Textbook/self-study	17	14.3
Didactic lectures	10	8.4
Presentation-based learning	6	5.0
Interactive classroom discussions	5	4.2

## Discussion

The goal of this study was to examine how a structured digital conversation tool could help second-year medical students learn more concepts in pathology and make learning easier. The results show that learning from textbooks is still associated with better academic performance in essay and short-answer assessments. However, using digital tools increased student interest and was particularly helpful for improving performance on MCQs.

The preference for digital modules among students aligns with findings by Sandars et al., who noted that modern learners, particularly those from the Net Generation, seek educational tools that match their digital lifestyle and provide instant access, clarity, and interactivity [22,23]. Our study reinforced this, as learners valued the self-paced structure and guided-flow design of the AI-based tool.

Interestingly, the digital group excelled in MCQs, consistent with Mayer et al., who emphasized that interactive learning platforms can enhance recognition- and recall-based learning [20]. Mayer's cognitive theory of multimedia learning supports the idea that combining visual and verbal information in a well-designed manner improves understanding [11].

Students who used textbooks performed better on tasks requiring explanation and connection of knowledge, such as essays and short answers. Cook et al. previously noted that e-learning improves accessibility, but traditional methods are often superior for long-form analytical tasks [24]. This aligns with Biggs et al., who found that deep learning develops more effectively through repeated use of in-depth resources like textbooks [25].

Despite the academic lag observed in digital learners for certain assessment types, the motivational benefits of conversational tools are substantial. Chi et al. emphasized that interactive engagement strategies foster critical thinking and facilitate the transition from passive to active learning [13]. Moreover, Vygotsky’s theory of social constructivism supports the idea that structured dialogue, even in digital form, can simulate the “zone of proximal development,” enabling learning beyond a student’s immediate capacity [12].

Our study’s results align with Laurillard's informal framework, which states that conceptual change occurs through iterative dialogue between learners and instructors or AI [19,26]. In this study, the AI-powered module acted as a surrogate teacher, providing guidance through structured questions and feedback. Regmi et al. found that students initially exposed to new educational technology were more engaged and curious [27]; however, they cautioned that such tools are less effective if not properly integrated into the educational framework.

Traditional learning remains prevalent in medical education, but integrating digital platforms as supplementary tools offers distinct benefits. Wong et al.'s review supports this blended approach, noting that e-learning is most effective when aligned with course objectives and assessment methods [28]. Our findings suggest that a hybrid model allows students to capitalize on the strengths of both approaches.

In conclusion, the digital module functioned not as a replacement but as a reinforcement of traditional learning, a view supported by Gaba et al., who advocate using technology as a complement rather than a substitute in medical education [29]. Our results confirm that such reinforcement is particularly effective during early stages of knowledge acquisition and for specific assessment formats.

**Figure 1:**
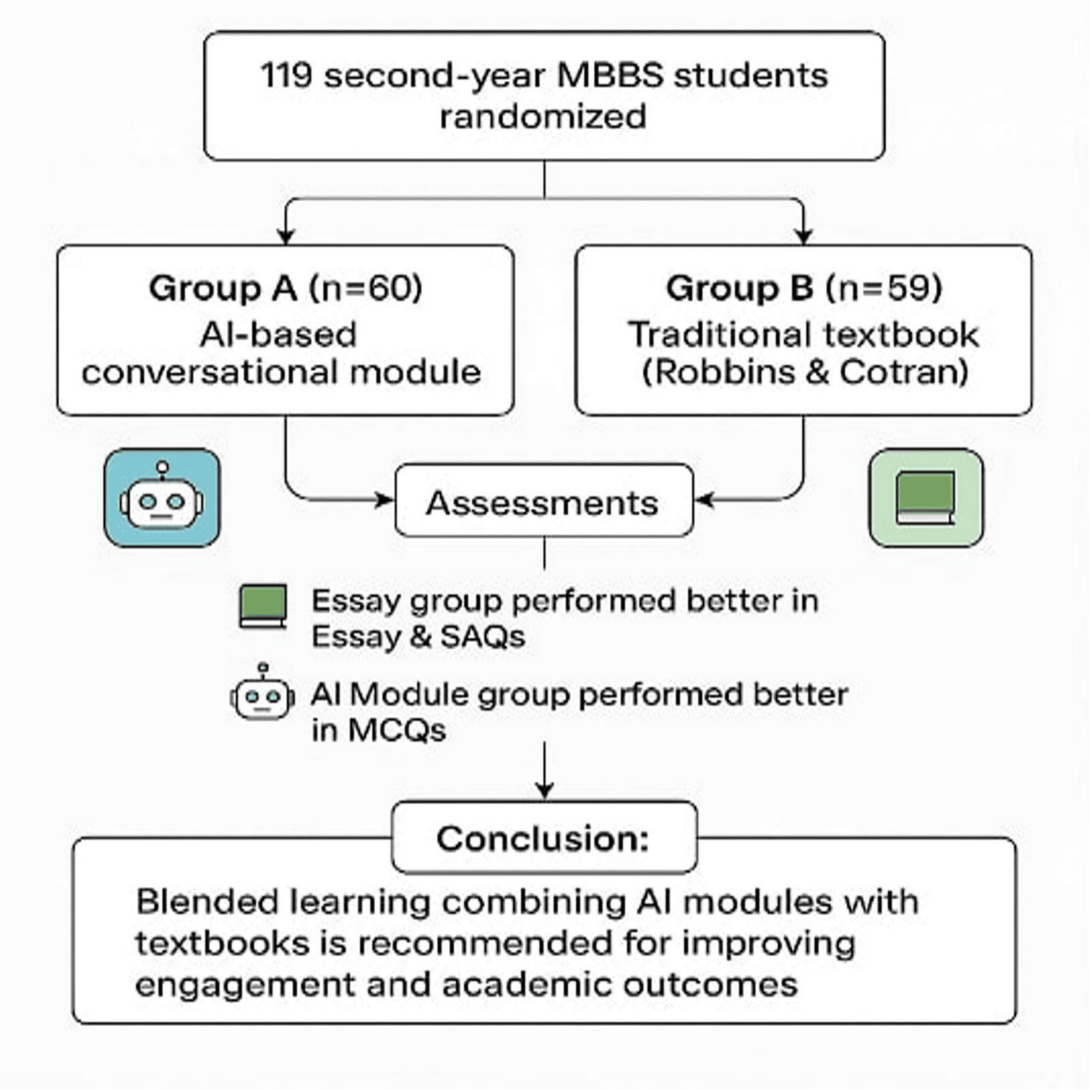
Graphical abstract illustrating the study design and key findings, recommending a blended learning approach SAQ, short-answer questions; MCQ, multiple-choice questions

Therefore, it is recommended that medical institutions adopt a blended learning model in which structured digital dialogue tools are strategically integrated with textbooks to enhance student engagement and performance [30]. Figure 1 presents the study design and summary of outcomes, highlighting the distinct impacts of the AI-based conversational module and the textbook-based approach. Students using textbooks performed better in essays and short-answer assessments, while AI module users excelled in MCQs. These findings suggest that both approaches enhance learning and that combining traditional resources with AI modules can maximize student engagement and outcomes.

Limitations

Every study has its limitations. In our study, the research was limited to a single institution, and the sample size was small, which may limit generalizability. The knowledge intervention was brief and focused on select pathology topics, and long-term knowledge retention was not assessed. Furthermore, the newly developed digital module may not have provided the same depth of content as traditional textbooks. To build on these findings, future research should include larger, multi-center sample sizes, extend study durations, and enhance the digital resources used in the research.

## Conclusions

This study demonstrates that while textbook-based learning produces higher exam scores overall, students also value the accessibility and clarity of structured digital modules. These tools are particularly valuable for promoting independent learning and engagement. A blended model that combines digital and traditional strategies may provide the best educational outcomes for medical students. As medical education continues to evolve, integrating well-designed, curriculum-aligned digital resources can support a richer and more personalized learning experience.
